# Synthesis of Zinc Oxide Nanoparticles with Bioflavonoid Rutin: Characterisation, Antioxidant and Antimicrobial Activities and In Vivo Cytotoxic Effects on *Artemia* Nauplii

**DOI:** 10.3390/antiox11101853

**Published:** 2022-09-20

**Authors:** Mansab Ali Saleemi, Batoul Alallam, Yoke Keong Yong, Vuanghao Lim

**Affiliations:** 1 Advanced Medical and Dental Institute, Universiti Sains Malaysia, Bertam, Kepala Batas 13200, Penang, Malaysia; 2 Department of Human Anatomy, Faculty of Medicine and Health Sciences, Universiti Putra Malaysia, Serdang 43400, Selangor, Malaysia

**Keywords:** ZnO nanoparticles, nanoformulation, green synthesis, cytotoxicity, reactive oxygen species

## Abstract

This study aims to synthesise zinc oxide nanoparticles with rutin (ZnO-R NPs) using a green synthesis approach and characterise the nanostructures for diverse biomedical applications. In this study, the optical and chemical properties of synthesised ZnO-R NPs were verified through Fourier transform infrared (FTIR) spectroscopy and ultraviolet-visible (UV-Vis) spectroscopy. The FTIR spectroscopy revealed a symmetric bending vibration peak of 460 cm^−1^ for ZnO-R NPs, whereas UV-Vis spectroscopy showed a distinct absorption band at 395 nm. Moreover, the oval-shaped morphology of ZnO-R NPs was verified through scanning electron microscopy and transmission electron microscopy. The synthesised nanoformulation revealed a wurtzite structure with a crystallite size of 13.22 nm; however, the zeta potential value was recorded as −8.50 ± 0.46 mV for ZnO-R NPs. According to an antioxidant study, ZnO-R NPs demonstrated lower free-radical scavenging activity than pure rutin. The cytotoxicity study was conducted using a human breast cancer cell line (MCF-7). In vitro analysis verified that ZnO-R NPs exhibited significantly higher anticancer and microbial growth inhibition activities than standard ZnO NPs (ZnO Std NPs) and pure rutin. In addition, ZnO-R NPs revealed a significantly lower IC_50_ value than the commercial ZnO Std NPs and pure rutin in MCF-7 cells (16.39 ± 6.03 μg/mL, 27 ± 0.91 μg/mL and 350 ± 30.1 μg/mL, respectively) after 48 h. However, synthesised ZnO-R NPs demonstrated no significant toxicity towards *Artemia* nauplii. These results highlight the synthesis of rutin-mediated ZnO NPs and their possible chemotherapeutic potential.

## 1. Introduction

The increasing number of functional nanomaterials being designed and developed has focused attention on their potential applications in various fields. A type of semiconductor known as nanocrystalline zinc oxide (ZnO) has a large band gap of 3.37 eV and a high exciton binding energy (60 meV) at room temperature [1]. Because of its various properties, such as its excellent thermal conductivity, ZnO is widely used in different applications, such as sensors [2], thin film devices [3,4] and solar cells [5,6]. The crystallinity and morphology of ZnO nanoparticles (NPs) are related to their chemical and physical properties [7]. In addition to being useful for reducing contaminants in the environment, ZnO NPs have a remarkable photocatalytic characteristic. The photocatalysis process is used to promote electrons (e^−^) from the valence to the conduction band and can generate a hole (h^+^) in the surface of ZnO through solar light irradiation. The resulting radical can then be used to break down pollutants. In addition, the process can migrate e^−^/h^+^ pairs to the ZnO surface [8,9]. ZnO is considered a better choice for photocatalysts than titanium dioxide when exposed to light because of its lower cost of production and nontoxicity [8,10]. Therefore, ZnO is a promising alternative to the traditional methods used to treat and disinfect wastewater.

In addition to being useful as an anticancer agent, ZnO NPs have other properties, such as antimicrobial and antioxidant capabilities [11,12,13]. Studies have revealed that the presence of ZnO in human cancer cells can induce various cellular responses, such as apoptosis and the degradation of cells. These findings indicate that the presence of ZnO in the cancer microenvironment can lead to the development of a preferentially acidic response [11,14,15]. The antibacterial activities of ZnO are attributed to its interactions with the cell surface and membrane of the bacteria [12]. Through the use of carrier proteins and ion channels, NPs can penetrate the cell wall and interact with cellular organelles, which can lead to the accumulation of reactive oxygen species (ROS). The interaction of ZnO with the cell membrane causes the lipid bilayer of bacterial and fungi to break down. This process allows the release of toxic substances from the cell. However, the size and shape of NPs are also key factors that can affect their ability to deliver toxic chemicals.

The use of ZnO has made tremendous progress in therapeutic areas such as bioimaging and drug delivery. Because of the increasing interest in the use of ZnO-based nanostructures in the development of drug delivery systems, preclinical research on this technology has also gained attention. Based on various studies, the US Food and Drug Administration has declared ZnO to be a safe inorganic compound; however, the use of ZnO as a therapeutic agent in drug delivery systems is still in its early stages. There have only been a few reports of the successful use of acid-degradable ZnO NPs for the delivery of chemotherapeutic drugs to cancer cells [14,16,17,18]. Moreover, several other studies have demonstrated that ZnO NPs loaded with curcumin [19,20,21], isotretinoin [22] or paclitaxel [23] have an anticancer effect. The bioflavonoid rutin is a compound found in various types of vegetables and plants. It is a powerful antioxidant that can help fight free radicals [24] and has the potential to be useful in treating various diseases and conditions, such as cancer. Its strong antioxidant, cytotoxic and antiproliferative activities can be attributed to its plant origins [25,26], and it prevents the development of human colon and lung carcinoma cells [24]. Despite an increasing understanding of rutin’s bacteria-killing capabilities, the development of metal oxide NPs with rutin has not gained much attention.

*Artemia* (brine shrimp) is zooplankton that is commonly used to feed the larval fishes [27]. One of the main characteristics of *Artemia* is their ability to adapt to various hypersaline environments, such as salt lakes and constructed salt pans. They play a vital role in the energy flow of nutrients into the marine environment [28,29]. It is possible to develop *Artemia*-based bioassays sustainably because the use of *Artemia* in toxicology poses a reasonable number of questions that can be answered, including practical considerations of laboratory culture and cyst attainment, ecological relevance, systematic use, and practical conditions of maintenance and sustainability of laboratory conditions for animal models [30]. A brine shrimp assay was developed as part of a study to find the cytotoxicity of synthesised ZnO-R NPs as a quick, simple and affordable screening for toxic substances. In this study, the rutin-mediated synthesis of ZnO NPs (ZnO-R NPs) was explored and the characterisation of synthesised NPs evaluated using scanning electron microscopy (SEM), transmission electron microscopy (TEM), Fourier transform infrared (FTIR) spectroscopy, X-ray diffraction (XRD), ultraviolet-visible (UV-Vis) spectroscopy and a Zetasizer. Studies indicate that the use of a bioactive flavonoid can reduce the toxicity of ZnO nanomaterial together with controlling the size and shape of the reaction and minimising nanomaterial impurities. To our knowledge, the in vivo cytotoxic effects of ZnO-R NPs on *Artemia* nauplii have not been explored. Therefore, in the present study, we investigated the antioxidant and microbial growth inhibition activity of ZnO-R NPs in different selected bacterial strains and studied the antiproliferative activity of synthesised ZnO NPs towards MCF-7 cells together with their in vivo toxic effects on *Artemia* nauplii. In addition, we explored the combination of rutin and the antitumorigenic and bacterial growth inhibition properties of ZnO to produce a hybrid (ZnO/rutin). The results indicated that the two compounds could produce a hybrid that has high bacterial growth inhibition and anticancer properties. Thus, the rutin-based synthesis of ZnO NPs could be used in various biomedical applications.

## 2. Materials and Methods

### 2.1. Chemicals

Zinc acetate dihydrate (EP grade) was supplied by Nacalai Tesque (Kyoto, Japan), and rutin and gentamycin sulfate were obtained from Biobasic (Markham, ON, Canada). The analysis and quantitative and qualitative measurements involved the following chemicals and media: commercial ZnO NPs (Sigma Aldrich, Saint Louis, MO, USA) (used as a standard) with a particle size of <100 nm, 2,2′-azino-bis(3-ethylbenzothiazoline-6-sulfonic acid) (ABTS) (Sigma Aldrich, Saint Louis, MO, USA), 2,2-diphenyl-1-picrylhydrazyl (DPPH) (Sigma Aldrich, Saint Louis, MO, USA), 3-(4,5-dimethylthiazol-2-yl)-2,5-diphenyltetrazolium bromide (MTT) (Sigma Aldrich, Saint Louis, MO, USA), dimethyl sulfoxide (DMSO) ≥ 99.5% GC (Sigma Aldrich, Saint Louis, MO, USA), Dulbecco’s modified eagle medium (DMEM) (Gibco, Grand Island, NY, USA), foetal bovine serum (FBS) (Gibco, Grand Island, NY, USA), Mueller Hinton agar (MHA) (Merck, Darmstadt, Germany), Mueller Hinton broth (MHB) (Merck, Darmstadt, Germany) and a human breast cancer cell line (MCF-7; American Type Culture Collection (ATCC)).

### 2.2. Synthesis and Characterisation of Zinc Oxide Nanoparticles

A biogenic synthesis of ZnO NPs was performed using an aqueous solution of rutin and zinc acetate dihydrate, as previously reported [31] with some modification. Briefly, 20 mL of zinc acetate (0.5 mM) was added to 20 mL of rutin after being dissolved in distilled water under magnetic stirring at room temperature. The reaction mixture was kept at room temperature for 2 h. The NPs were condensed and purified using centrifugation at a rate of 2000× *g* for 10 min. The NPs were then rinsed with distilled water three times and calcinated at 550 °C for 1 h. The synthesised ZnO NPs were suspended in distilled water with a stock concentration of 1 mg/mL and sonicated for 5 min before each analysis.

Initially, the synthesised ZnO-R NPs were analysed using a quantitative technique UV-Vis spectroscopy (Shimadzu, Kyoto, Japan) at a range of 200–800 nm, and SEM (Hitachi-Regulus, Tokyo, Japan) was then applied to determine their morphology. For this purpose, the samples were directly placed on a carbon tape without coatings. The particle dimensions were derived from a series of SEM images. Average measurements were used to approximate the obtained dimensions. To study the structure of the ZnO-R NPs, they were suspended in distilled water, sonicated and then coated onto a copper grid. They were then dried and examined using TEM (Zeiss, Oberkochen, Germany). Moreover, XRD (Empyrean, Malvern Panalytical, Malvern, UK) analysis was performed to determine the presence of ZnO NPs and phase composition along with the crystallite size and strains using Co-Kα1 radiation (λ = 1.54056 A). The current used to test the powder was 40 kV/30 mA in the 2θ range from 20° to 90° using Cu-Kα1 radiation, and the crystallite size and lattice strain of the synthesised NPs were calculated using the Williamson–Hall method [32].

FTIR was used to identify the various functional groups and phytochemical constituents that play a role in the reduction and stabilisation of NPs. A Perkin Elmer 100 spectrophotometer (Norwalk, CT, USA) was used to perform FTIR in the attenuated total reflectance (ATR) mode. The results of this study were observed within the range of 4000–400 cm^−1^. The size distribution and zeta potential were evaluated using a Zetasizer Nano ZS (Empyrean, Malvern Panalytical, Malvern, UK).

### 2.3. Antioxidant Activities

The antioxidant activity was examined using a DPPH in vitro assay. A two-fold serial dilution was prepared (3.906, 7.813, 15.625, 31.250, 62.500, 125, 250 and 500 µg/mL) for the samples using a stock solution (1 mg/mL).

The antioxidant activity of the synthesised ZnO-R NPs and standard ZnO NPs (ZnO Std NPs) was investigated, as previously reported [33] with modification. Briefly, 150 μL of DPPH (0.2 mM) was applied to 50 μL of samples at different concentrations (3.906–500 µg/mL) in methanol (100%). The colour change was monitored using a microplate reader (BioTek, Santa Clara, CA, USA) at 517 nm in the dark at 37 °C. Rutin and butylated hydroxyanisole (BHA) were used as controls in this experiment. The inhibitory effects were determined using the following equation [34]:

Radical scavenging (%) = [*Ab_control_* − *Ab_sample_*/*Ab_control_* × 100%] (1)

where *Ab_control_* is the absorbance of the DPPH solution and *Ab_sample_* is the absorbance of the DPPH solution with the absorbance of the sample.

However, another method for determining the antioxidant activity of synthesised NPs is to identify ABTS radical scavenging activity, as previously reported [34] with some modification. In brief, a working solution of ABTS cation radicals was prepared by mixing 7 mM of ABTS solution and 2.45 mM of potassium persulphate at a 1:1 ratio. The mixture was left to stand for 16 h in the dark. The solution was further diluted with 99.5% ethanol, and absorbance was checked at 734 nm using a microplate reader (BioTek, Santa Clara, CA, USA). Individual wells were pipetted with the ABTS working solution (180 µL) and sample (20 µL). The mixture was incubated at room temperature for 6 min. The absorbance at a wavelength of 734 nm was then measured, with rutin and BHA as controls. The inhibitory effects were determined using the following equation [34]:

Radical scavenging (%) = [*OD_control_* − OD_sample_/*OD_control_* × 100%] (2)

where *OD_control_* is the ABTS working reagent absorbance with ethanol and *OD_sample_* is the absorbance of the ABTS working reagent with the sample.

### 2.4. Bacterial Growth Inhibition of the Prepared Zinc Oxide Rutin Nanoparticles

The inhibitory effect was explored using a disc diffusion method. Subsequently, a broth microdilution assay was applied to determine the minimum inhibitory concentration (MIC) and minimum bactericidal concentration (MBC).

#### 2.4.1. Microbial Strains

In this experimental study, microbial strains such as *Escherichia coli* (ATCC 25922), *Klebsiella pneumoniae* (BAA-1705), *Staphylococcus aureus* (ATCC 25923) and methicillin-resistant *S. aureus* (ATCC 33591) were used to observe the inhibitory effects of the synthesised ZnO-R NPs. The bacterial cultures were collected from the Microbiology Laboratory of Clinical Trial Complex, Advanced Medical and Dental Institute, Universiti Sains Malaysia, Kepala Batas, Pulau Pinang, Malaysia. The bacterial isolates were preserved in glycerol (10%) at −80 °C until further use.

#### 2.4.2. Preparation of Bacterial Cultures and Samples

A single colony of bacteria was transferred to 10 mL of MHB from the stock culture. The culture was then suspended at 37 °C for 24 h. Using a UV-Vis spectrophotometer, dilutions of the culture with MHB equivalent to 1–2 × 10^8^ CFU/mL were prepared and quantified at 600 nm. The absorbance should be 0.1, which corresponds to a concentration of 10^8^ CFU/mL. Subsequently, 5 mg of each sample was dissolved in 1 mL of distilled water. The samples were sonicated further for 15 min using an ultrasonic cleaner (WUC-A10H, Wise Clean, Sonic Wise, CA, USA). The samples were then sterile filtered before being used.

#### 2.4.3. Disc Diffusion Method

The disc dilution method was used to assess the inhibitory effects of ZnO-R NPs against several bacterial strains. The assay was performed according to the Clinical and Laboratory Standards Institute (CLSI) (M02-A11). In this approach, a zone of inhibition produced by the identified pathogens was determined using MHA. The agar was placed into a Petri dish (Bio-Rad, Hercules, CA, USA), and the plates were left to solidify for 5–10 min. A concentration of bacterial cells (1–2  ×  10^8^ CFU/mL) was then spread on the solidified agar plates [35]. Later, a sterile disc (ø = 9 mm) was soaked with 30 µL of the samples. The positive control was gentamicin and ciprofloxacin, and the negative control was distilled water. Finally, the Petri dishes were incubated for 24 h at 37 °C, and a clear inhibition zone was measured and expressed in mm.

#### 2.4.4. Broth Microdilution Assay

A broth microdilution technique was used to determine the MIC and MBC. This method was performed as described by CLSI (M07-A9) with some modifications. The sample was serially diluted in 96-well plates with flat bottoms from 0.9 to 1000 μg/mL. A test sample (20 μL) and bacterial inoculum suspension (180 μL) were added to each well. The positive control was gentamicin and ciprofloxacin, and the negative control was distilled water. The plates were then kept in an incubator overnight at 37 °C. Subsequently, each well received 50 μL of MTT solution (0.5 mg/mL), and the plate was incubated for 30 min. The presence of live bacteria was observed by the darkening of the colour. The MBC values were determined by streaking 10 µL of the nonpurple samples from the wells onto the MHA and then incubating these samples at 37 °C. The MBC was evaluated after 24 h to differentiate between the bacteriostatic and bactericidal agents from the test samples [36].

### 2.5. Cell Cultivation

The cytotoxicity of the test samples was investigated using the MCF-7 cell line. MCF-7 cells were cultured in DMEM, which was supplemented with penicillin and streptomycin (1%) together with 10% FBS. In this study, the cells were cultured at 37 °C with carbon dioxide (CO_2_; 5%), and the growth of cells was routinely observed under the microscope. The cells were grown in a 25 cm^2^ cell culture flask and then trypsinised to extract them once they reached confluence (~80%) for the MTT experiment.

#### Cytotoxicity Test (MTT Assay)

The cytotoxicity of the synthesised ZnO-R NPs, ZnO Std NPs and rutin in MCF-7 was assessed using an MTT test [34]. Briefly, the cells were cultivated at a density of (1.2 × 10^6^ cells/mL) in 96-well microplates and then kept in a 37 °C incubator with CO_2_ (5%). The wells were filled with fresh DMEM medium containing ZnO-R NPs at final concentrations of 3.6–200 µg/mL. The cells were allowed to grow for 24 or 48 h before adding 10 µL of MTT (5 mg/mL). The medium was replaced with DMSO (10%) to dissolve the resulting crystals of formazan after 4 h of incubation. A microplate reader (BioTek, USA) was applied to measure the optical density at 570 nm. The viability of cells (%) was determined using the following formula [34]:

Cell viability (%) = *OD_sample_*/*OD_control_* × 100% (3)

where *OD_control_* is the absorbance of the untreated cells and *OD_sample_* is the sample absorbance of the treated cells.

### 2.6. Lethality of the Artemia Nauplii Assay

The acute toxicity of various concentrations of ZnO-R NPs, ZnO Std NPs and rutin on *Artemia* nauplii growth, survival and mortality was examined. *Artemia franciscana* cysts were first hydrated for 12 h at 4 °C in distilled water. Using a Buchner funnel, the sinking cysts were collected and rinsed with cold distilled water. Approximately 3 g of precleaned cysts were incubated in 1.5 L of seawater (3%, *w*/*v*) with graduations at 30 ± 1 °C. A constant fluorescent lamp (1500 lux daylight) was used, and aeration was provided by a short pipe flowing from an aquarium air pump to the bottom of the hatching device. *Artemia* larvae hatched in 24 h under these conditions.

The experiment was conducted in a 12-well plate with nauplii less than 24 h old and continued for 4, 8 and 24 h. Each well was filled with 1 mL of seawater and control samples (without NPs). Approximately 15 nauplii were transferred with the addition of the desired concentration of the samples (0.781–100 µg/mL). After incubating the nauplii for 24 h, the percentages of mortality and half-maximal lethal concentration (LC_50_) values were calculated and compared with the control. Potassium dichromate was used as a positive control. An inverted phase-contrast microscope was used to examine the morphological differences between the treated and untreated nauplii.

### 2.7. Statistical Analysis

The values were expressed as mean ± standard deviation (SD) for three independent experiments. An analysis of variance was performed to evaluate and compare data using Minitab software, followed by a Tukey’s post hoc multiple comparison test, with *p* < 0.05 considered a significant difference.

## 3. Results and Discussion

Rutin, a pure bioflavonoid, was used in the synthesis of ZnO NPs. The colour change from a light yellow to a white precipitate confirmed the synthesis of ZnO NPs [37]. Plant extract-assisted synthesised ZnO NPs from *Plectranthus amboinicus* [38] and *Jacaranda mimosifolia* [39] generated a white precipitate, which matched our findings. The reduction of zinc salt to ZnO NPs can be attributed to the presence of certain molecules and functional groups in rutin, which was confirmed by FTIR studies.

### 3.1. Characterisation of Zinc Oxide Rutin Nanoparticles

#### 3.1.1. Structural Characterisation

**Figure 1:**
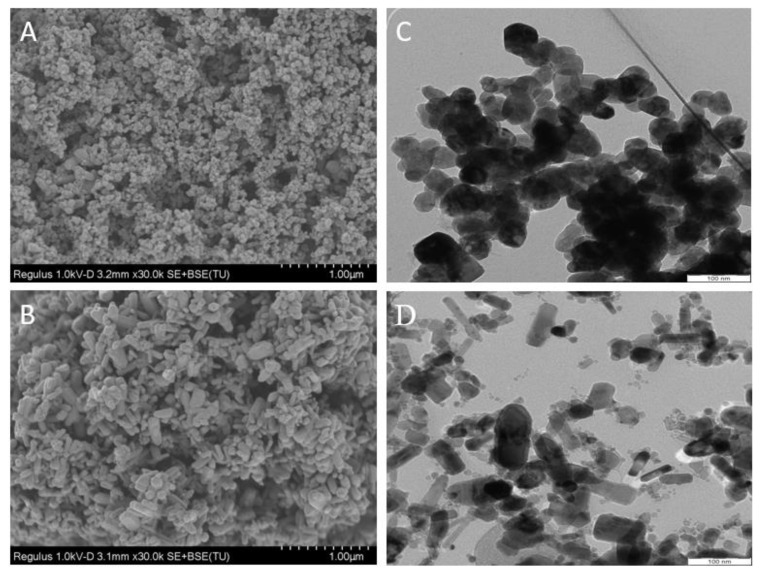
SEM images of (**A**) ZnO-R NPs and (**B**) ZnO Std NPs. TEM images of (**C**) ZnO-R NPs and (**D**) ZnO Std NPs.

**Figure 2:**
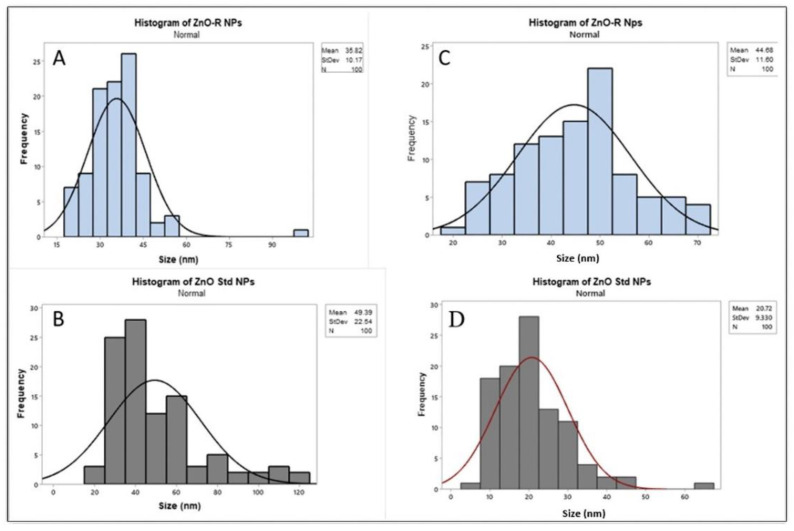
Histogram from the SEM of (**A**) ZnO-R NPs and (**B**) ZnO Std NPs. Histogram from the TEM of (**C**) ZnO-R NPs and (**D**) ZnO Std NPs.

The nanofabrication of ZnO NPs with rutin was determined using TEM and SEM images, as shown in Figure 1. After analysing the SEM images, NPs with a slight ovoid form were identified (Figure 1A). The diameter of the ZnO-R NPs determined through SEM was recorded as 35.82 ± 10.17 nm (Figure 2A), whereas the diameter of the ZnO Std NPs was determined as 49.39 ± 22.54 nm (Figure 2B). According to the TEM images, ZnO-R NPs also exhibited spherical-shaped morphologies (Figure 1C), with a diameter of 44.68 ± 11.60 nm (Figure 2C), whereas ZnO Std NPs displayed a rod-shaped morphology (Figure 1D), with a diameter of 20.72 ± 9.330 nm (Figure 2D). The particle size of the ZnO-R NPs was in the range of 20–40 nm (Figure 2A,C). Previous studies have used different methods to synthesise rutin-conjugated ZnO NPs in various forms and sizes [24,40,41]. For instance, one study reported that the morphology of synthesised copper(II) oxide (CuO) NPs with chitosan using rutin was spherical [42]. The interaction of chitosan with CuO NPs may result in the colloidal nature of the synthesised nanocomposite. Another study synthesised ZnO NPs using pure rutin, observing that the morphology was rod shaped [24], which is consistent with this study. In one study, the morphology of biologically produced ZnO NPs was described as rod shaped with a size range of 80–130 nm [43]. However, researchers have also used an isolated flavonoid derived from the medicinal plant *Combretum ovalifolium* to biosynthesise ZnO NPs using a precipitation process [44]. The flavonoid was used as a reducing agent for a zinc precursor (zinc acetate dihydrate), and the flavonoid-mediated ZnO NPs were found to be fibre-shaped and single-crystalline, with an average diameter of 31.24 nm [44]. Another study used zinc nitrate as a precursor, reporting the microwave-induced production of ZnO nanorods as a quercetin carrier [40]. The rutin-conjugated ZnO nanorods revealed a hexagonal wurtzite structure with a diameter of 100–200 nm. The shape and size of ZnO NPs are determined by a variety of kinetic parameters, including the originating plant species, reactant concentration and pH. In this study, we employed the green method with a one-pot reaction using pure rutin as a reducing agent. The fabrication of ZnO NPs using pure rutin can be explained by their ability to bioaccumulate metal ions along with their bioreduction and stabilisation during the process. In general, the hydroxyl (OH) groups in rutin lead to the development of a [Zn(OH)_4_]^2−^ complex, which is a critical growth unit for ZnO NPs. The zinc–base ratio, which affects both the nucleation and subsequent growth of ZnO nanostructures, can then be modulated to cause preferential growth along one crystalline plane [45].

#### 3.1.2. Chemical Characterisation

**Figure 3:**
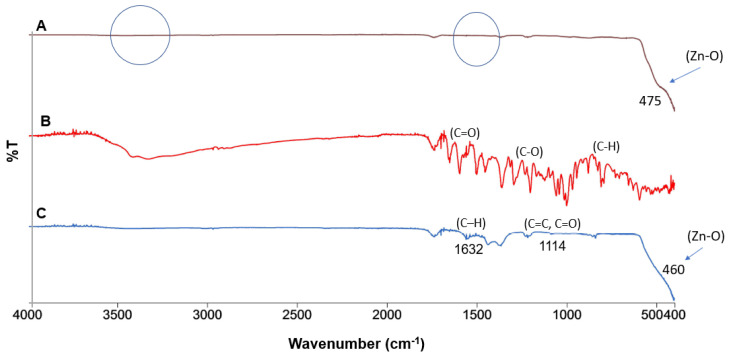
ATR–FTIR spectra of (**A**) ZnO Std NPs, (**B**) rutin and (**C**) ZnO-R NPs.

Figure 3 depicts the ATR–FTIR spectrum of the ZnO Std NPs, ZnO-R NPs and rutin bioflavonoid. The transmissions at 460 and 475 cm^−1^ were found to be produced by a symmetric bending vibration of Zn–O in the ZnO-R NPs and ZnO Std NPs, respectively [24,39,46]. There were no bands at 1700 or 3500 cm^−1^, which are represented by the surface H–OH bending vibration and O–H stretching vibration, respectively, suggesting that the ZnO surface had no hydroxyl-group adsorption, exhibiting the formation of ZnO rather than Zn(OH)_2_. In the pure rutin spectrum, the transmission peaks observed at 1690, 1235 and 850 cm^−1^ were assigned to the C=O stretching vibration of aryl ketones, C–O phenol stretching vibration and C–H aromatic hydrocarbon bending vibration, respectively. However, low-frequency peaks occurred in the spectrum of the ZnO-R NPs at 1632–1114 cm^−1^, corresponding to the C=C, C=O and C–H stretching of the –COOH group [47]. Therefore, the reduction in OH frequency verified the bonding between rutin and ZnO. The presence of the aforementioned functional groups (primarily derived from the rutin bioflavonoid) was supported by the other FTIR spectra (Figure 3). However, rutin is a polyphenolic compound with a high concentration of phenolic acids [24,41]. The presence of certain functional groups in the rutin-containing compounds may help in the reduction, stabilisation and capping of ZnO NPs [24,48].

#### 3.1.3. Physical Characterisation

**Figure 4:**
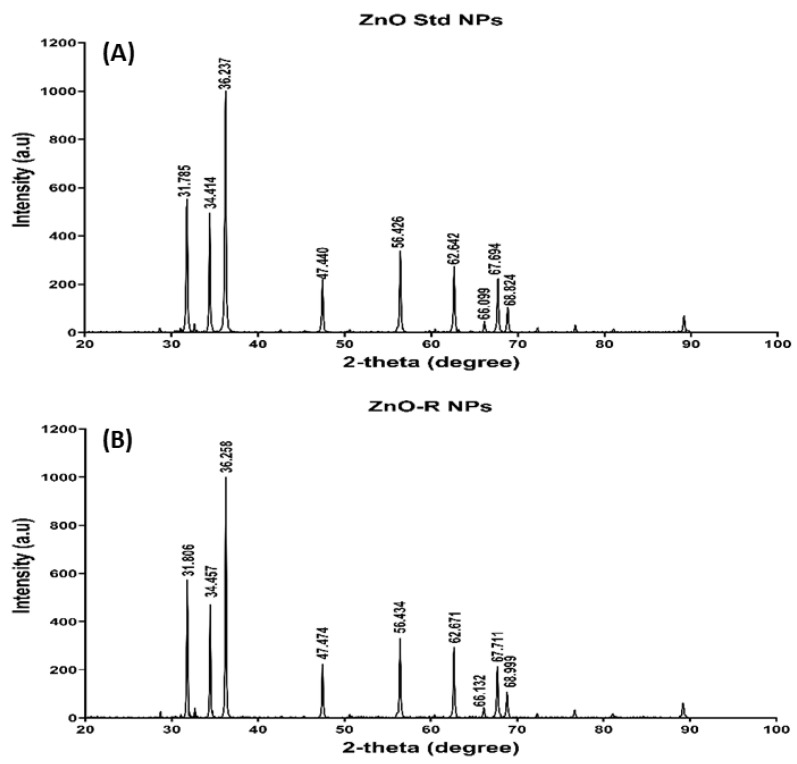
XRD of (**A**) ZnO Std NPs and (**B**) ZnO-R NPs.

In general, the crystalline structure of ZnO NPs can be determined through an XRD analysis. In this study, the physical characterisation of the synthesised nanoformulation was performed using an XRD pattern, as illustrated in Figure 4. All the diffraction peaks were found at 31.785, 34.414, 36.237, 47.440, 56.426, 62.642, 67.694 and 68.824, which corresponds to (110), (002), (101), (102), (110), (103), (200) and (112) orientation planes [49], indicating a typical zincite ZnO structure diffraction (hexagonal phase, space group P63*mc*, with lattice constants *a* = 3.24890 A°, *c* = 5.20620 A°, *Z* = 2) [50]. These peaks are consistent with those found in the hexagonal phase of the wurtzite structure of ZnO Std NPs. The synthesised NPs were strongly crystallised, indicated by the sharp diffraction, high intensity and narrow width peaks shown in Figure 4 [50]. The polycrystalline structure of the synthesised NPs was suggested by the strong and largest peak at (101). The nanoformulations produced by Jeyaleela et al. had diffraction peaks in the (100), (002) and (101) planes, which were closer to the reported wurtzite structure values [44]. Crystallite sizes of 11.05 and 13.22 nm were obtained for ZnO Std NPs and ZnO-R NPs, respectively. However, the average size of rutin-mediated ZnO NPs was reported as 31.24 nm [44,51].

The hydrodynamic size of the ZnO Std NPs and ZnO-R NPs was 909.00 ± 65.18 nm and 804.67 ± 68.86 nm, respectively. Compared with the size acquired through the TEM analysis, the size appeared to be greater under dynamic light scattering apparatus. This might result from the ZnO NPs’ considerable hydration or solvation in water. The zeta potential values of the ZnO Std NPs, ZnO-R NPs and pure rutin were recorded as 2.76 ± 0.19, −8.50 ± 0.46 and −8.37 ± 0.16 mV, respectively. Generally, wurtzite-type ZnO NPs have positive surface charges [52]. The accumulation of rutin on the surface of ZnO NPs could explain the significant change in the charge intensity of the outer layers of ZnO. Moreover, the polydispersity index values of the ZnO Std NPs and ZnO-R NPs were 0.412 ± 0.039 and 0.336 ± 0.051, respectively. The aggregation of particles causes the broad particle size distribution, and this aggregation is caused by the polarity and electrostatic attraction of ZnO NPs [53].

#### 3.1.4. Optical Characterisation

**Figure 5:**
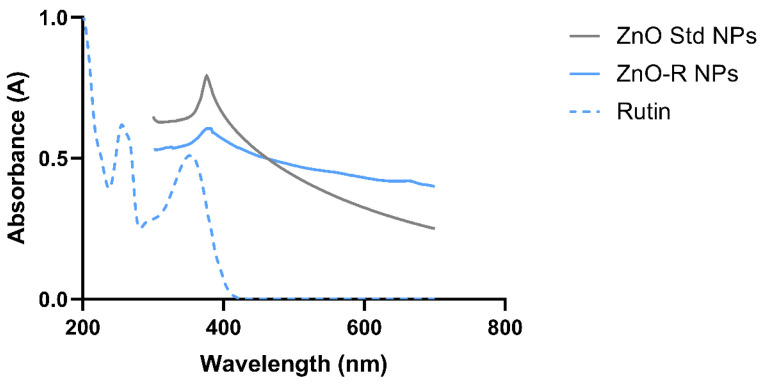
UV-Vis absorption spectra of ZnO Std NPs, ZnO-R NPs and rutin.

The UV-Vis spectra of the synthesised nanoformulation were recorded to investigate the optical properties of ZnO-R NPs (Figure 5). In this study, ZnO Std NPs displayed a strong absorption band at 385 nm, which is inconsistent with another study that stated that ZnO NPs exhibited a distinctive broad absorption peak between 330 and 460 nm [54]. Pure rutin exhibited λ_max_ at 275 and 388 nm, which was then shifted to 394 nm after the formation of the ZnO NPs. The UV-Vis spectra of flavonoids also revealed two absorption maxima, one at 245–285 nm (band-II for a ring B cinnamoyl system) and the other at 300–400 nm (band-I for a ring A benzoyl system) [44]. A peak was attained at 395 nm for the ZnO-R NPs, which verified the successful fabrication of ZnO NPs with a pure bioflavonoid compound (rutin). This absorption peak could be attributed to ZnO’s intrinsic band-gap absorption caused by electron transitions from the valence to the conduction band (O2p→Zn3d) when exposed to UV light [55]. This provides evidence that rutin is involved in the reduction of Zn^2+^ to Zn^0^ [56,57].

### 3.2. Antioxidant Activity

The excessive formation of reactive oxygen species (ROS) through metabolic processes in the human body may result in cellular damage [24]. ROS are known to play a significant role in the development of human disorders [48]. Antioxidants serve as free-radical scavengers, which reduce the possibility of chronic disease development [41]. The antioxidant effect of the ZnO Std NPs, ZnO-R NPs and pure rutin was analysed using DPPH and ABTS.

**Figure 6:**
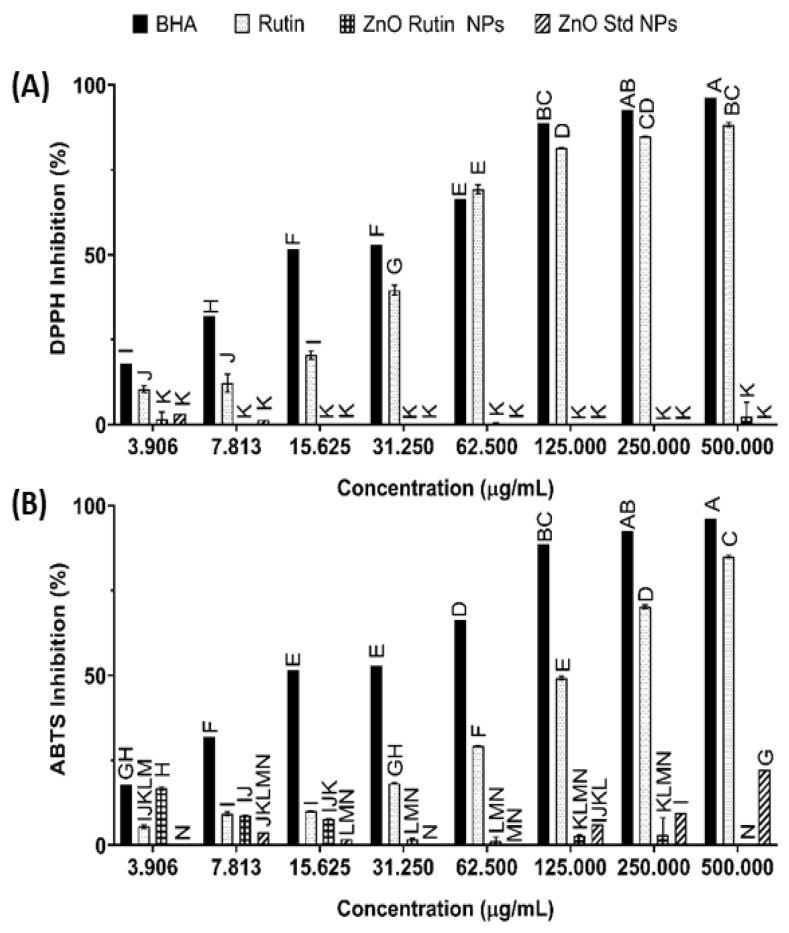
(**A**) DPPH and (**B**) ABTS assays. Samples with different letters differ significantly (*p* < 0.05, mean ± SD, *n* = 3).

Figure 6A presents the effects of the DPPH inhibition activity of the synthesised nanoformulation compared with that of BHA. The synthesised nanoformulation exhibited an inhibitory effect on DPPH radicals. The DPPH activity of rutin exhibited an IC_50_ value of 122.27 ± 1.25 µg/mL. BHA demonstrated a significantly lower IC_50_ value of 20.04 ± 2.01 µg/mL, whereas ZnO Std NPs and ZnO-R NPs showed a significantly higher IC_50_ value than BHA and pure rutin (>500 µg/mL). The capacity of rutin and BHA to scavenge DPPH significantly increased in a dose-dependent manner; nevertheless, BHA exhibited a significantly higher activity than rutin at different concentrations, but it was not as high at 62.5 µg/mL. Rutin was significantly more effective as an antioxidant agent than the ZnO NPs at all concentrations. In addition to having a π system, DPPH molecules also have an unpaired electron in the nitrogen atom. The molar absorptivity increases when the aromatic ring is substituted. This effect is also apparent if the length of the substituent is increased. The reaction between DPPH and the antioxidant occurs when the H–atom from the donor atom is transferred to an odd electron in the radical [58]. The change in the colour of the sample is caused by the conversion of free radicals to nonfree radicals [59]. The ZnO-R NPs and ZnO Std NPs in this study were not rich in hydrogen, as demonstrated by the FTIR results, which explains their low antioxidant activity. In one study, ZnO NPs produced using a *R. graveolens* stem bark extract showed concentration-dependent DPPH antioxidant activity, which is consistent with our findings [56]. In another study, ZnO NPs derived from *Curcuma longa* rhizomes demonstrated that they were effective hydroxyl radical scavengers, scavenging over 70% of hydroxyl radicals at 200 µg/mL, which is slightly less than for ascorbic acid [60]. Phenolic is an essential compound for antioxidant properties and is present in ZnO NPs [24,61].

The ABTS assay highlights the efficiency of antioxidants in scavenging ABTS and forming ABTS^•+^. Antioxidants act as agents for hydrogen donating [62]. Rutin, with an IC_50_ value of 166.72 ± 1.54 µg/mL, had significantly higher antioxidant activity than ZnO NPs with a concentration of 3.9 µg/mL. BHA exhibited significantly lower antioxidant activity than rutin at concentrations ranging from 3.9 to 500 µg/mL. Both BHA and rutin demonstrated a dose-dependent radical scavenging inhibition effect (Figure 6B). The reduction in ABTS was significantly enhanced when using higher concentrations. BHA and rutin scavenging activity increased as concentrations increased from 3.9 to 500 µg/mL.

### 3.3. Microbial Growth Inhibition

In this study, ZnO Std NPs revealed an MIC of 500 µg/mL against both *E. coli* and methicillin-resistant *S. aureus* (Table 1). ZnO-R NPs and rutin did not demonstrate any activity in relation to Gram-negative or Gram-positive bacteria. Similarly, neither the ZnO NPs nor rutin exhibited MBC values. The inhibitory effect of ZnO particles is usually inversely related to their size. Therefore, ZnO nanostructures should exhibit greater antibacterial activity than bulk ZnO [63,64]. ZnO NPs were shown to have an MIC of 1 mg/mL against *S. aureus* [65]. In general, Gram-negative bacterial species are less sensitive to ZnO than Gram-positive bacterial strains. This could result from the differences in the nature and organisation of microbial cell walls. As Gram-positive bacteria have a thick cell wall, they are more prone to producing large amounts of peptidoglycan. However, Gram-negative bacteria have a complex structure and are less likely to produce large amounts of peptidoglycan. The outer membrane of a cell contains high levels of protein, lipids and polysaccharides, and the outer surface of the periplasmic region is covered by a thin layer of peptidoglycan, which is surrounded by another membrane [66].

In the present study, the size of the inhibitory zones varied according to the type of bacteria (Table 1). *E. coli* exhibited less susceptibility to the synthesised ZnO-R NPs (9.376 ± 0.07 mm), mainly because it has a thin layer of peptidoglycan. ZnO Std NPs revealed an inhibition zone against *E. coli* (10.597 ± 0.08 mm) almost two- to three-fold smaller than those of gentamicin (22.87 ± 0.30 mm) and ciprofloxacin (32.25 ± 0.12 mm). However, it was also observed that the MIC and MBC values of gentamicin were higher than those of ciprofloxacin, whereas the zone of inhibition of gentamicin was smaller for different selected bacteria. The inhibition zone of ZnO-R NPs against *E. coli* and *S. aureus* was found to be substantially smaller than that of ZnO Std NPs.

According to previous studies, the effect of ZnO NPs on bacteria is related to the production of ROS and zinc ions in the NPs, which can adhere to the negatively charged surface of bacterial membranes, inducing cell death through cellular material leakage [24,38]. ZnO NPs combined with rutin demonstrated bactericidal effects against *S. aureus* (9.563 ± 0.26). However, the antibacterial inhibitory activity of rutin-mediated ZnO NPs was higher than that of pure rutin or ZnO Std NPs [24]. Another study demonstrated that 57% of *E. coli* bacterial growth was recorded when the microbial isolates were exposed to a ZnO/flavonoid nanocomplex (80 μg/mL), whereas only 41–43% of growth inhibition was recorded when they were exposed to ZnO (48 μg/mL) or flavonoids (32 μg/mL) separately [40]. Thus, the chemical reaction of rutin makes bacterial cells sensitive to ZnO NPs.

### 3.4. Anticancer Activity

**Figure 7:**
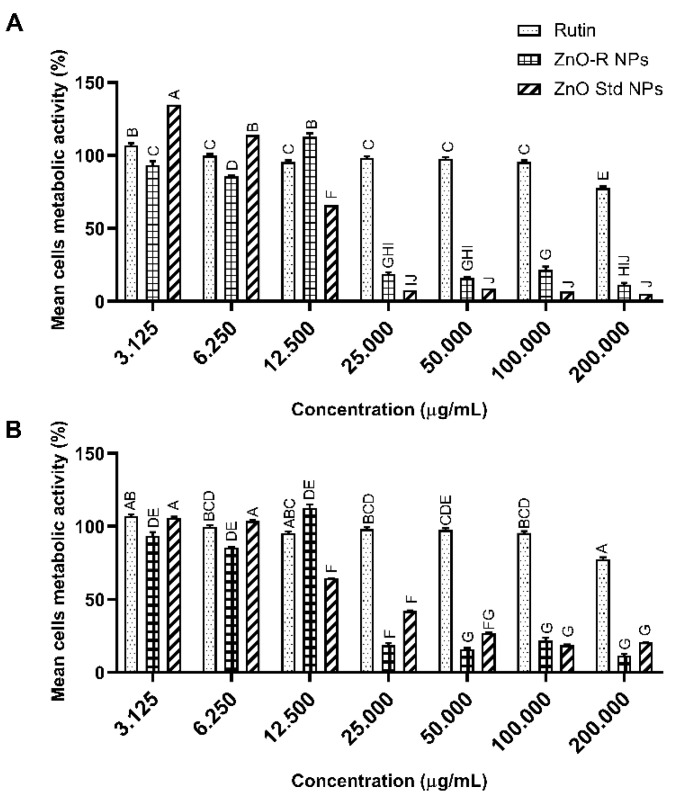
Relative MCF-7 cell viability 24 h (**A**) and 48 h (**B**) post-treatment at various concentrations of ZnO Std NPs, ZnO-R NPs and rutin (mean ± SD; *n* = 3). Letters denote significance; groups that do not share letters are significantly different (*p* < 0.05, *n* = 3).

The in vitro antiproliferative activity of ZnO-R NPs, ZnO Std NPs and rutin in MCF-7 was studied using an MTT assay. The cells were subjected to various concentrations of rutin and the synthesised nanoformulation for 24 and 48 h. As shown in Figure 7, ZnO-R NPs exhibited dose- and time-dependent anticancer activity with a concentration of 3.125–200 μg/mL. ZnO-R NPs were found to have a higher antiproliferative effect than pure rutin or ZnO Std NPs. After 24 h, the IC_50_ value of the ZnO-R NPs was reported to be 23.51 ± 0.12 μg/mL, whereas the IC_50_ value of the pure rutin treated cells was 346.97 ± 31.83 μg/mL. Moreover, exposure to ZnO Std NPs resulted in an IC_50_ value of 14.08 ± 0.91 μg/mL after 24 h. The IC_50_ value of the ZnO-R NPs was recorded as 16.39 ± 6.03 μg/mL, with the IC_50_ value of ZnO Std NPs and pure rutin observed as 27 ± 0.91 μg/mL and 350 ± 30.1 μg/mL, respectively, at 48 h. Compared with ZnO, rutin exhibited less cytotoxicity and poor in vitro bioavailability because of the lack of a carrier–deliverer for the target cells [24]. It is also believed that the use of ZnO NPs could lead to the development of effective antitumour agents [44]. The band gap and catalytic activity of these materials can help their toxic response in biological systems [15,17]. Although the actual mechanism underlying the cytotoxic response of ZnO NPs remains undetermined, several theories have been proposed. The intracellular release of dissolved zinc ions, together with the production of ROS, is the principal mechanism behind ZnO NP cytotoxicity. This is caused by the binary response, which includes the proinflammatory reaction of cells to ZnO NPs and the unique surface characteristic that allows ZnO NPs to function as a redox system [58,59].

**Figure 8:**
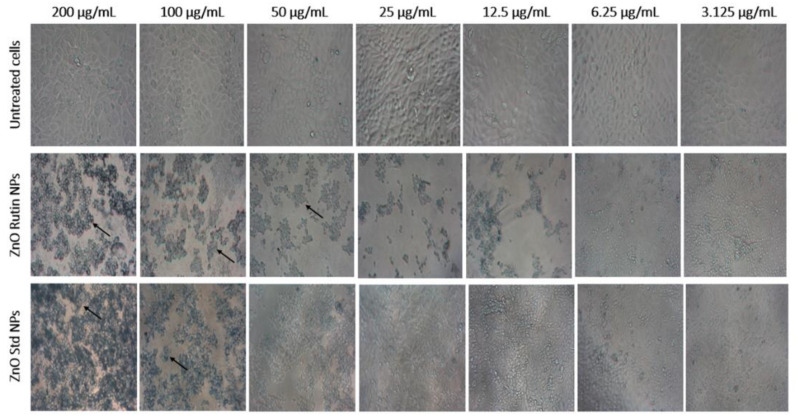
Bright-field micrographs obtained at 20× magnification of cells treated at various concentrations of ZnO NPs.

In addition, bright-field micrographs of cells exposed to ZnO-R NPs and ZnO Std NPs were used to verify the anticancer potential. The presence of apoptotic bodies, cell shrinkage and the blebbing of cell membranes were all clear indicators of the cytotoxicity of the NPs (Figure 8) [40]. In comparison to cells exposed to ZnO Std NPs, the cytotoxic effects of cells exposed to ZnO-R NPs were found to be greater. Cytotoxicity is the key factor for anticancer drugs. In this study, synthesised ZnO-R NPs were found to cause considerable cytotoxicity in MCF-7 cells, suggesting that more research on the anticancer mechanism of these nanoformulations is required.

### 3.5. Lethality in Artemia Nauplii

**Figure 9:**
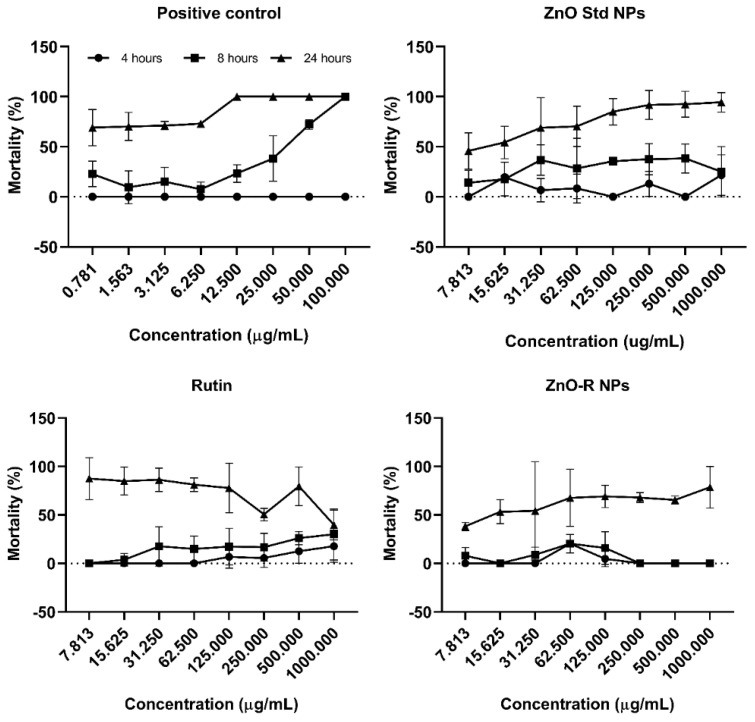
Mortality rates of *Artemia* nauplii at different concentrations of NPs and rutin.

The in vivo cytotoxicity of different nanoformulations was determined using a brine shrimp lethality assay. The results of the experiments revealed that feeding deprivation did not cause death in *Artemia* nauplii even after 24 h. The positive control demonstrated no mortality in 4 h; however, a concentration-dependent effect revealed a significant mortality at 8 and 24 h, as shown in Figure 9. In 8 h, the mortality rate was almost 30% when the concentration of ≤6.25 µg/mL was used. However, the mortality rate was significantly different when the concentration of samples was increased from 6.25 to 100 µg/mL. A 100% mortality rate was observed at a concentration of 100 µg/mL. Moreover, the lethal effect of samples was more obvious after 24 h of exposure. The average mortality rate was determined to be 69.03% at a concentration of 0.78 μg/mL and 100% at 100 μg/mL, revealing an LC_50_ value of 0.34 μg/mL.

In the ZnO Std NP-treated group, the mortality rate increased with the increasing NP concentration and an extended incubation time (*p* < 0.05). In addition, no significant difference was identified in the mortality rate between all the studied concentrations at 4 and 8 h. At 4 h, the mortality rate ranged from 0% (0.78 μg/mL) to 21.67% (1000 μg/mL), and at 8 h, it ranged from 14.07% (0.78 μg/mL) to 25% (1000 μg/mL). Moreover, the mortality rate ranged from 45.83% (0.78 μg/mL) to 94.44% (1000 μg/mL) at 24 h (LC_50_ = 10.93 μg/mL).

It was observed that ZnO-R NPs resulted in a significantly different mortality rate to that of ZnO Std NPs. At 0.78 μg/mL, the lethal effects were observed as 0%, 7.94% and 38.16% at 4, 8 and 24 h, respectively. This increased to 78.57% at 1000 μg/mL after 24 h. Thus, the LC_50_ of the ZnO-R NPs was higher than that of the ZnO Std NPs (LC_50_ = 15.64 μg/mL).

At 24 h, the temporal pattern of the rutin’s lethal effects was identical to those of the ZnO Std NPs and ZnO-R NPs. Within the same concentration gradient, however, no mortalities were recorded between 4 and 8 h. Although zinc is an essential component of most biological organisms, excessive exposure to it can cause cellular damage. Research on the toxic effects of ZnO NPs showed that the higher cytotoxicity of nanoparticles compared to its bulk formulation was due to increased solubilisation [62,67]. By contrast, the results of studies demonstrated that the toxicity of nZnO aggregates was not caused by particle dissolution. Soluble Zn(^2+^) was able to reduce the toxicity of these aggregates to different organisms more than nZnO [68,69,70].

**Figure 10:**
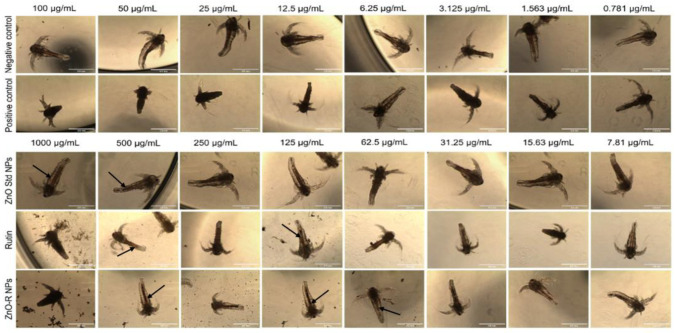
Morphology of the treated and untreated *Artemia* nauplii after treatment with the prepared samples.

Figure 10 shows the aggregation of ZnO NPs inside the gut of *Artemia* nauplii. The filter-feeding behaviour of *Artemia* is nonselective; they consume any particle smaller than 50 μm. The amount consumed by each individual animal at varying concentrations has an impact on the aggregation level. As illustrated in Figure 10, the guts of the animals in the control group were empty (no signs of aggregation; the mouthparts and gut region appeared transparent), but the guts of the animals in the treatment group were completely full of NPs. The images also verified that *Artemia* nauplii were able to eliminate the ingested NPs. Moreover, no significant change in the size of *Artemia* nauplii was observed compared with that of the negative control. Regarding the positive control, their size was significantly smaller at concentrations higher than 25 μg/mL (*p* < 0.005).

## 4. Conclusions

The current research used pure bioflavonoid rutin to create environmentally safe ZnO NPs. The results revealed that rutin successfully mediated the synthesis of ZnO NPs using a green chemistry approach. In this study, the optical, structural and physicochemical properties of ZnO-R NPs were investigated. For this purpose, FTIR spectroscopy and UV-Vis spectroscopy were used to assess the chemical and optical characteristics of these synthesised nanoformulations. The oval-shaped morphology of ZnO-R NPs was determined through a microscopic examination; a wurtzite structure with crystallite sizes of 11.05 and 13.22 nm for ZnO Std NPs and ZnO-R NPs, respectively, was also observed. The diameter of the ZnO-R NPs determined through SEM was recorded as 35.82 ± 10.17 nm, whereas the diameter of the ZnO Std NPs was determined as 49.39 ± 22.54 nm. In addition, the antioxidant results showed that BHA demonstrated a significantly lower IC_50_ value of 20.04 ± 2.01 µg/mL, whereas ZnO Std NPs and ZnO-R NPs showed a significantly higher IC_50_ value than BHA and pure rutin (>500 µg/mL). We also found that ZnO-R NPs have the ability to suppress bacterial growth and act as anticancer agents, exhibiting dose- and time-dependent anticancer activity at different concentrations. Moreover, ZnO-R NPs have a significant antiproliferative effect than pure rutin and ZnO Std NPs, suggesting that more research on the anticancer mechanisms underlying these nanoformulations is required. Furthermore, no significant difference in toxicity was found between ZnO-R NPs and ZnO Std NPs towards *Artemia* nauplii. Therefore, we anticipate that the produced ZnO-R NPs might be employed as nanobioflavonoids in the pharmacological and biomedical fields.

## Figures and Tables

**Table 1:** MIC, MBC and the zone of inhibition of ZnO Std NPs, ZnO-R NPs and rutin.

Bacteria	Gentamicin	Ciprofloxacin	Rutin	ZnO-R NPs	ZnO Std NPs
		MIC (µg/mL)			
*E. coli*	1.95	0.98	na	na	500
*K. pneumoniae*	15.63	0.98	na	na	na
*MRSA*	7.81	1.95	na	na	500
*S. aureus*	0.98	0.98	na	na	na
		MBC (µg/mL)			
*E. coli*	7.81	1.95	na	na	na
*K. pneumoniae*	31.25	1.95	na	na	na
*MRSA*	31.25	3.91	na	na	na
*S. aureus*	1.95	1.95	na	na	na
		Inhibition zone (mm)			
*E. coli*	22.87 ± 0.30	32.25 ± 0.12	na	9.376 ± 0.07	10.597 ± 0.08
*K. pneumoniae*	11.63 ± 0.10	19.27 ± 0.22	na	na	9.597 ± 0.36
*MRSA*	11.2 ± 0.06	23.4 ± 0.36	na	na	na
*S. aureus*	15.7 ± 0.04	24.86 ± 0.05	na	9.563 ± 0.26	na

na: no activity.

## Data Availability

All data is comprised within this manuscript.
